# Supplementary material for: Formal modeling of a causal consistent distributed system and verification of its history via model checking using colored Petri net
Source: PeerJ Comput Sci. 2025 Jul 7;11:e2995. doi: 10.7717/peerj-cs.2995 (PMC12453694; doi:10.7717/peerj-cs.2995)
Supplement: Supplemental Information 2 [file peerj-cs-11-2995-s002.docx]

1: // Algorithm for group g's members $P_{i}(i=1,2\ldots,N)$
2: Initialization of the Fidge's vector clocks of all group g's members

3: $V_{i}^{g}\left[ j \right]:=0 \left( i,j=1,2\ldots,N \right)$

4: CO-multicast of message $m$ to g's members by process $P_{i}$:

5: 1. $V_{i}^{g}[i]:=V_{i}^{g}[i]+1$

6: 2. B-multicast $(<V_{i}^{g},m>)$
7: When $P_{j}$B-delivers $(<V_{i}^{g},m>)$ from $P_{i}$ $(j\neq i)$, with $g=group(m)$ :

8: 1. It places $<V_{i}^{g},m>$ in its hold-back queue
9: 2. Wait until $V_{i}^{g}[i]=V_{j}^{g}[i]+1$ and $V_{i}^{g}[k]\leq V_{j}^{g}[k](k\neq i)$;
10: 3. CO-deliver m; // after removing $<V_{i}^{g},m>$ from the hold-back queue

11: 4. $V_{j}^{g}[i]:=V_{j}^{g}[i]+1$;
