# Supplementary material for: Formal modeling of a causal consistent distributed system and verification of its history via model checking using colored Petri net
Source: PeerJ Comput Sci. 2025 Jul 7;11:e2995. doi: 10.7717/peerj-cs.2995 (PMC12453694; doi:10.7717/peerj-cs.2995)
Supplement: Supplemental Information 3 [file peerj-cs-11-2995-s003.docx]

CPN Tools state space report for:

/cygdrive/C/Users/ASUS/Desktop/causalConcistency57-CaseStudy1.cpn

Report generated: Tue Apr 22 23:01:43 2025

Statistics

-------------------------------------------------------------

State Space

Nodes: 46

Arcs: 72

Secs: 0

Status: Full

Scc Graph

Nodes: 46

Arcs: 72

Secs: 0

Home Properties

-------------------------------------------------------------

Home Markings

None

Liveness Properties

-------------------------------------------------------------

Dead Markings

8 [9,46,45,39,38,...]

Dead Transition Instances

None

Live Transition Instances

None

Fairness Properties

-------------------------------------------------------------

No infinite occurrence sequences.
