# Supplementary material for: Formal modeling of a causal consistent distributed system and verification of its history via model checking using colored Petri net
Source: PeerJ Comput Sci. 2025 Jul 7;11:e2995. doi: 10.7717/peerj-cs.2995 (PMC12453694; doi:10.7717/peerj-cs.2995)
Supplement: Supplemental Information 5 [file peerj-cs-11-2995-s005.docx]

CPN Tools state space report for:

/cygdrive/C/Users/ASUS/Desktop/causalConcistency57-CaseStudy3.cpn

Report generated: Fri May 16 21:22:11 2025

Statistics

------------------------------------------------------------------------

State Space

Nodes: 23081

Arcs: 62140

Secs: 77

Status: Full

Scc Graph

Nodes: 23081

Arcs: 62140

Secs: 1

Home Properties

------------------------------------------------------------------------

Home Markings

None

Liveness Properties

------------------------------------------------------------------------

Dead Markings

5062 [9999,9993,9992,998,9978,...]

Dead Transition Instances

None

Live Transition Instances

None

Fairness Properties

------------------------------------------------------------------------

No infinite occurrence sequences.
