# Supplementary material for: Formal modeling of a causal consistent distributed system and verification of its history via model checking using colored Petri net
Source: PeerJ Comput Sci. 2025 Jul 7;11:e2995. doi: 10.7717/peerj-cs.2995 (PMC12453694; doi:10.7717/peerj-cs.2995)
Supplement: Supplemental Information 6 [file peerj-cs-11-2995-s006.docx]

CPN Tools state space report for:

/cygdrive/C/Users/ASUS/Desktop/causalConcistency57-CaseStudy4.cpn

Report generated: Fri May 16 21:47:50 2025

Statistics

------------------------------------------------------------------------

State Space

Nodes: 17867

Arcs: 43886

Secs: 15

Status: Full

Scc Graph

Nodes: 17867

Arcs: 43886

Secs: 1

Home Properties

------------------------------------------------------------------------

Home Markings

None

Liveness Properties

------------------------------------------------------------------------

Dead Markings

7223 [9995,9994,9992,9990,9989,...]

Dead Transition Instances

None

Live Transition Instances

None

Fairness Properties

------------------------------------------------------------------------

No infinite occurrence sequences.
